# A global long-term daily reanalysis of reference evapotranspiration for drought and food-security monitoring

**DOI:** 10.1038/s41597-023-02648-4

**Published:** 2023-10-27

**Authors:** Mike Hobbins, Timen Jansma, Daniel P. Sarmiento, Amy McNally, Tamuka Magadzire, Harikishan Jayanthi, Will Turner, Andrew Hoell, Greg Husak, Gabriel Senay, Olena Boiko, Michael Budde, Pamella Mogane, Candida F. Dewes

**Affiliations:** 1 University of Colorado, Cooperative Institute for Research in Environmental Sciences, Boulder, CO USA; 2 National Oceanic and Atmospheric Administration-Physical Sciences Laboratory, Boulder, CO USA; 3 University of Groningen, Groningen, The Netherlands; 4 National Aeronautics and Space Administration-Goddard Space Flight Center, Greenbelt, MD USA; 5 Science Application International Corporation, Reston, VA USA; 6 University of California Santa Barbara, Department of Geography, Climate Hazards Center, Santa Barbara, CA USA; 7 Famine Early Warning Systems Network, Washington, D.C., USA; 8 ASRC Federal Data Solutions, contractor to the U.S. Geological Survey Earth Resources Observation and Science Center, work performed under USGS contract 140G0119C0001, EROS Center, Sioux Falls, SD USA; 9 United States Geological Survey Earth Resources Observation and Science Center, Sioux Falls, SD USA; 10 University of Colorado-North Central Climate Adaptation Science Center, Boulder, CO USA; 11 KBR, Inc. under contract to the U.S. Geological Survey Earth Resources Observation and Science Center (140G0121D0001), EROS Center, Sioux Falls, SD USA; 12 University of Colorado, National Snow and Ice Data Center, Boulder, CO USA

**Keywords:** Hydrology, Natural hazards, Water resources, Developing world, Agriculture

## Abstract

NOAA has developed a global reference evapotranspiration (ET_0_) reanalysis using the UN Food and Agriculture Organization formulation (FAO-56) of the Penman-Monteith equation forced by MERRA phase 2 (MERRA2) meteorological and radiative drivers. The NOAA ET_0_ reanalysis is provided daily from January 1, 1980 to the near-present at a resolution of 0.5° latitude × 0.625° longitude. The reanalysis is verified against station data across southern Africa, a region presenting both significant challenges regarding hydroclimatic variability and observational quantity and quality and significant potential benefits to food-insecure populations. These data are generated from observations from the Southern African Science Service Centre for Climate Change and Adaptive Land Management (SASSCAL) network. We further verified globally against spatially distributed ET_0_ derived from two reanalyses–the Global Data Assimilation System (GDAS) and Princeton Global Forcing (PGF)–and these verifications produced similar results, yet demonstrated wide regional and seasonal differences. We also present cases that verify the operational applicability of the reanalysis in long-established drought, famine, crop- and pastoral-stress metrics, and in predictability assessments of drought forecasts.

## Background & Summary

While agricultural and meteorological droughts may be defined as anomalously low soil moisture and precipitation, respectively, they are both revealed as a surplus in evaporative demand (E^0^), which is defined as the upper, energetic limit on evaporation from the surface^1^. Evaporative demand is an atmospheric state for which a useful analogy is as a measure of the thirst of the atmosphere: it physically integrates radiative and advective forcing variabilities and, further, reflects water availability through land surface–atmosphere feedbacks that affect partitioning of the available energy at the surface into latent and sensible heat fluxes^2,3^. The relationship between actual evapotranspiration (ET; the supply of moisture from the surface to the atmosphere) and evaporative demand (the demand for that moisture in the atmosphere) reflects surface moisture availability in plants, soil, or water bodies. In general, in the energy-limited (humid) range of the hydroclimatic spectrum, E_0_ drives actual ET in a parallel direction, while in the water-limited (dry) range, E_0_ is driven by actual ET in complementary directions^2^.

While the primary metrics of evaporative demand—pan evaporation, potential evaporation, and reference evapotranspiration (ET_0_)—differ in their surface assumptions and whether they are observed (pan evaporation) or synthesised (potential evaporation and ET_0_)—they all estimate the amount of surface water that would evaporate into the overpassing air under prevailing advective and radiative conditions and assuming sufficient non-limiting surface moisture availability. The concept has traditionally been used in irrigation scheduling and estimating crop water requirements^4^, but also has utility in water-management modelling, as an input to land-surface models (LSMs), in crop-stress modelling, especially in regions where parameterizations of soil- and plant-water availability are uncertain or observational data are lacking, and in drought monitoring^5^, where it is used an independent drought indicator either by itself^6,7^ or in combination with other fluxes^8,9^.

Here we describe the development of the new NOAA ET_0_ dataset, funded by the Famine Early Warning Systems Network (FEWS NET) with the aim of improving the ET_0_ used in their existing drought and famine early warning efforts, including the Water Requirement Satisfaction Index (WRSI^10,11^ and USGS Water Points^12^). These currently rely upon a global ET_0_ dataset^13^ derived from NOAA’s Global Data Assimilation System (GDAS^14^), which is available at a six-hourly time step with single-day latency, but at a coarse spatial resolution (1°). GDAS ET_0_ spans 2001 to the present, which is short relative to satellite data availability and to the recommended 30+ years for calculation of standardised indices^15^. Further, GDAS is subject to constant improvements and adjustments to input data streams, analysis algorithms, and resolutions: these changes can negatively affect its utility in ongoing food-security monitoring and planning (see Methods). These difficulties in placing GDAS-derived water requirement anomalies in proper historical contexts underscore the need for temporal coherence in modelling across the entire period of record. Therefore, a clear need exists for an improved dataset—one with more consistent inputs, finer spatial resolution, and a longer period of record. The new ET_0_ reanalysis should hew as closely as possible to a sound physical understanding of the flux, meet international scientific and operational standards, replace and improve upon existing datasets, and accurately replicate its relationship to drought.

The specific metric of evaporative demand used herein is the FAO-56 formulation^4^ of ET_0_, which uses the Penman-Monteith^16^ equation to estimate the ET flux under given prevailing atmospheric conditions and carefully specified surface conditions: a reference crop of either 0.12-m grass or 0.5-m alfalfa that is well-watered, actively growing, and completely shading the ground. This is the worldwide standard for a fully physical ET_0_ estimate. For input data, we use drivers from the Modern-Era Retrospective analysis for Research and Applications, Version 2 (MERRA2^17^).

The primary operational limitation of using MERRA2 is its multi-week latency, which limits its applicability in real-time drought monitoring and famine early warning. In order to mitigate the effects of this limitation on the users of the data, NOAA also uses preliminary MERRA2 data that are available two weeks before the MERRA2 general release to generate a gap-filling daily ET_0_ series. Including this series at the leading edge of the MERRA2-forced ET_0_ reduces the data latency to about 10 days. ET_0_ estimated from preliminary data are overwritten when the officially released driver data become available. Note that this preliminary data is not considered in any of the verification or case studies presented here.

Following is a description of the development of the new ET_0_ dataset: a long-term, daily, global, fully physical representation of evaporative demand useful to drought and food-security monitoring operations. In “Methods,” we describe how ET_0_ is computed and how the MERRA2 forcing data has improved both spatial and temporal characteristics over GDAS. In “Technical Validation,” we demonstrate the dataset utility and accuracy and constrain the uncertainty of its estimates by verifying the new MERRA2-based ET_0_ dataset against ground-truth ET_0_ observations derived from the Southern African Science Service Centre for Climate Change and Adaptive Land Management (SASSCAL) station network and against the original, GDAS-based dataset and the Princeton Global Forcing v3 reanalysis (PGF^18^; http://hydrology.princeton.edu/data.pgf.php). We limit our station-based verification to southern Africa to address both significant data availability challenges and potential food security benefits. In “Use Cases,” we demonstrate drought-monitoring applications of the new ET_0_ dataset. Note that this Data Descriptor presents a static description of the dataset, peer reviewed in 2022.

## Methods

### Calculation of reference evapotranspiration (ET_0_)

The FAO-56 formulation^4^ provides a widely accepted estimate of ET_0_ derived from the Penman–Monteith equation^16^. It assumes that the surface is amply watered, actively growing reference crop whose canopy completely covers the ground with a fixed SW albedo of 0.23. The central equation (Eq. (1)) takes the form of a weighted combination of two driving terms: a radiative term (1st RHS term) that reflects the availability of energy at the evaporating surface and an advective term (2nd RHS term) that reflects the ability of the overpassing air to absorb and carry away evaporated moisture. The equation for daily ET_0_ [mm d^−1^] takes the form:

1 $$E{T}_{0}=\frac{0.408\Delta }{\Delta +\gamma \left(1+{C}_{d}{U}_{2}\right)}\left(\left(1-a\right){R}_{d}-{R}_{nl}-G\right)+\frac{\gamma }{\Delta +\gamma \left(1+{C}_{d}{U}_{2}\right)}\frac{{C}_{n}}{0.5\left({T}_{max}+{T}_{min}\right)+273}{U}_{2}\left({e}_{s}-{e}_{a}\right)$$

where T_max_ and T_min_ are respectively the maximum and minimum daily 2-m air temperatures [K], with an average of T; ∆ and γ are respectively the slope of the saturation vapour pressure-temperature curve at T and the psychrometric constant [both kPa °C^−1^]; U_2_ is the 2-m wind speed [m s^−1^]; ɑ is the (dimensionless) shortwave albedo, fixed for the assumed surface conditions at 0.23; R_d_ is the downwards shortwave radiation at the surface [W m^−2^], R_nl_ is the net upward longwave radiation at the surface [W m^−2^]; G is the ground heat flux [W m^−2^], assumed to be negligible on a daily basis; and e_s_ and e_a_ are the saturation vapour pressure and the mean actual vapour pressure at 1.5- to 2.5-m heights [both kPa], respectively. C_n_ is the numerator constant [K mm s^3^ Mg^−1^ d^−1^] and C_d_ is the denominator constant [s m^−1^], which take values specific to reference crop and timescale of estimation. The multiplier 0.408 represents the inverse of the latent heat of vaporisation [in kg J^−1^] and converts ET_0_ from an energy flux to a daily depth.

We estimate ET_0_ for two different reference crops: ET_0_ for a 0.5-m high alfalfa crop is denoted ETrs; ET_0_ for a short-grass crop (0.12 m) is denoted ETos. For ETrs, C_n_ = 1600 and C_d_ = 0.38; for ETos, C_n_ = 900 and C_d_ = 0.34.

In Eq. (1) the dependent variables ∆, γ, e_s_, and e_a_ are estimated as follows:

2 $$\Delta =2503\frac{exp\left(\frac{17.27\left(0.5\left({T}_{max}+{T}_{min}\right)-273.15\right)}{0.5\left({T}_{max}+{T}_{min}\right)-35.85}\right)}{{\left(0.5\left({T}_{max}+{T}_{min}\right)-35.85\right)}^{2}},$$

3 $$\gamma =0.000665P,$$

4 $${e}_{s}=0.5\ast 0.6108\left(exp\left(\frac{17.27({T}_{max}-273.15)}{{T}_{max}-35.85}\right)+exp\left(\frac{17.27({T}_{min}-273.15)}{{T}_{min}-35.85}\right)\right),$$

and

5 $${e}_{a}=\frac{qP}{0.622+0.378q}.$$

Estimating e_a_ in this manner (Eq. 5) hews as closely as possible to the top preference for humidity estimation in the FAO-56 protocols^4^. Estimation of R_nl_ is also detailed in that documentation, but it may be summarised as the following function of air temperature, e_s_, and relative cloudiness:

6 $${R}_{nl}=\sigma \left[0.5\left({T}_{max}^{4}+{T}_{min}^{4}\right)\left(0.34-0.14\sqrt{{e}_{a}}\right)\left(1.35\frac{{R}_{d}}{{R}_{so}}-0.35\right)\right],$$

where σ is the Stefan-Boltzmann constant [4.903 × 10^−9^ MJ K^−4^ m^−2^ d^−1^], R_so_ is the clear-sky SW downwelling radiation [W m^−2^], and the relative shortwave radiation (R_d_/R_so_) is limited to ≤1.0.

ET_0_ can be therefore calculated for each day and point in space from five input variables (drivers): R_d_, T, surface atmospheric pressure P [kPa], U_2_, and specific humidity at 2 m q [kg/kg].

### Selection of appropriate driver data set

For drivers, we use the MERRA2 dataset, which assimilates remotely sensed observations^17,19^ and is the only modern reanalysis to assimilate aerosol data^20,21^, which is crucial for capturing long-term tendencies in surface fluxes such as ET_0_ that are driven by shortwave and longwave radiation and in which there are strong trends^22^. MERRA2 has the benefit of being updated routinely but, unlike other operational products, has been reprocessed to maintain a consistent time series when updates to data streams and algorithms have been introduced. MERRA2 provides all five input variables required to estimate FAO-56 ET_0_ with complete global coverage at a 0.5° latitudinal × 0.625° longitudinal resolution from January 1, 1980 to within a few weeks of the present. While the accuracy of many MERRA2 variables has been assessed^17,23^, this is not the case for its estimation of ET_0_.

There are some known sources of uncertainty with the MERRA2 data. T estimates in MERRA2 are significantly correlated (r > 0.3) with rainfall across the tropics, including the Africa Sahel^24^. It follows that errors in rainfall–themselves likely due to lack of station data in this region^25^–will be propagated to errors in T, both in terms of anomalies and longer-term trends.

### Comparison with existing ET_0_ formulation used by FEWS NET

The ET_0_ reanalysis currently used by FEWS NET is driven by GDAS^13^. One goal of this new ET_0_ reanalysis is to improve on the GDAS-driven ET_0_. Here we discuss the primary differences between these two ET_0_ reanalyses relating both to specifications of the driving datasets and to the assumptions inherent in the respective estimation procedures used, as shown in Table 1.

**Table 1:** Differences between the various ET_0_-forcing global reanalyses: the MERRA2, GDAS (whose ET_0_ is being replaced by that forced by MERRA2), and the PGF reanalysis (whose ET_0_ is compared to the MERRA2-forced ET_0_ in Fig. 2 and Section 3 of Technical Validation).

	MERRA2	GDAS	Princeton v3
Spatial resolution	0.5° lat × 0.625° long	1°	0.25°
Temporal resolution	Hourly	6-hourly	3-hourly
Data latency	~2-week (preliminary), 1-month latency (final)	<1 day	N/A
Period of record	January 1, 1980 to near-present	2001 to near-present	1948–2016
Wind speed estimation	orthogonal 2-m U and V vectors	orthogonal 10-m U and V vectors, scaled to 2 m assuming a logarithmic vertical wind profile	
Net radiative flux estimation (R_n_ = R_d_ − R_u_ + L_d_ − L_u_)	strict FAO-56 formulation for reference conditions: • R_d_ from MERRA2 • R_u_ = ɑR_d_ • ɑ = 0.23 • L_d_ − L_u_ = f (e_s_, R_d_), Eq. (6)	residual of the four radiative fluxes from GDAS, with implicitly variable ɑ: • R_d_ − R_u_ + L_d_ – L_u_	
Citations	^17,23^	^14^	^18^

As an operational dataset, GDAS is subject to constant improvements and adjustments to input data streams, analysis algorithms, and resolutions; it is not reprocessed after these changes, leading potentially to downstream impacts on outputs or derived quantities–in our case, ET_0_ (see Technical Validation). For example, in May, 2007, the Spectral Statistical Interpolation 3-D variational data assimilation system in GDAS was replaced with the new Gridpoint Statistical Interpolation system (GSI^26^), but this change was not applied retrospectively. This was only one of a few changes to GDAS modelling in 2007 (https://www.emc.ncep.noaa.gov/gmb/STATS/html/model_changes.html): in late-September, the GSI was adjusted to assimilate wind data from new sources; in December, an upgrade to the Global Forecast System (GFS) ensemble was made but, again, not retrospectively. In the case of the GDAS-derived ET_0_ in West Africa, for example, this led to jumps from the earlier (pre-2007) to later periods (post-2007) in both means and coefficients of variation of annual ET_0_. Analysis of these changes (not shown) has indicated a transition in ET_0_ between the two periods that starts on or around 2007 and runs until around 2010. Other changes in spatial resolution often lead to “blocky” artefacts in temporally aggregated gridded surfaces.

While the new, MERRA2-based ET_0_ dataset is at a coarser timestep, this daily resolution is sufficient to meet all requirements of ET_0_ users in the drought and famine early warning communities. Note that the assumption of zero G in Eq. (1) is physically tenable (and approved by FAO-56) only at a daily resolution. The data latency—how long users have to wait for drivers to become available—is an important characteristic for monitoring applications. MERRA2 is primarily a research-oriented dataset, so the disadvantage of its multi-week latency is mitigated by use of preliminary data later over-written. The longer period of record of the MERRA2-forced ET_0_ allows for more robust assessments of current conditions with respect to long-term climatology. Further, longer climatologies better represent mean conditions as they are not overly influenced by individual instances of long-cycle climatic variations, such as the El Niño Southern Oscillation (ENSO), the Southern Oscillation Index (SOI), or the Pacific Decadal Oscillation (PDO). As an operational example of this advantage, the Evaporative Demand Drought Index (EDDI^6,7^) shown in the test-case below (Usage Notes 1a) requires at least a 30-year record against which to compare current conditions, and so could not be generated from GDAS-forced ET_0_. We note that other, long-term global reanalyses of evaporative demand tend either to contain discontinuities (such as the GDAS reanalysis we seek to replace here) or not be available in operational timeframes (such as the four ET_0_ reanalyses derived from the Penman-Monteith equation at https://wci.earth2observe.eu/ that are only available to 2012 or 2014) and are therefore unsuitable for use in operational food-security monitoring.

There are three differences in the parameterizations used to generate ET_0_ between the datasets. First, we estimate daily ET_0_ using the strict daily form of the FAO-56 formulation^4^ forced by daily maximum and minimum temperatures, whereas the GDAS dataset we seek to replace took a hybrid approach in which the hourly form of the ET_0_ formulation is driven by 6-hourly temperatures. Second, the 2-m wind speed estimation height of the MERRA2-forced ET_0_ is the FAO-56 standard, whereas the conversion of the 10-m winds to a 2-m equivalent in the GDAS formulation requires the assumption of a logarithmic wind profile. Finally, the most significant scientific difference is in the estimation of the net surface radiation (R_n_). The GDAS-derived ET_0_ uses a radiation budget for the surface conditions prescribed within the GDAS model, including a spatially variable albedo, whereas the MERRA2-based ET_0_ estimates the net radiation flux (R_nl_) in strict adherence to the FAO-56 parameterization^4^ of the Penman-Monteith equation^16^, which estimates R_nl_ assuming the reference surface conditions specified for ET_0_. Note that there are instances at high latitudes in winter that net radiation can be negative (i.e., net long wave upwards exceeds net shortwave radiation downwards): in these areas, ET_0_ may be negative, though very small. We do not artificially constrain such pixels to positive values.

 In summary, our new, MERRA2-driven ET_0_ dataset improves upon the GDAS-driven dataset by providing a longer time series, a higher spatial resolution, an improved driver suite, and a closer fidelity to both the FAO-56 formulation and the concept of reference evapotranspiration. It also provides a second formulation to bracket estimation uncertainty. The goal of this paper is not to assess different formulations but rather present the new ET_0_ dataset as a replacement for the GDAS-driven ET_0_. As part of the verification process (Technical Validation, part 3), we compare the two ET_0_ reanalyses.

## Data Records

The NOAA ET_0_ archive^27^ is available at the USGS Sciencebase (10.5066/P9IIQMV1) and is updated as soon as the MERRA2 drivers^28,29^ are released on NASA’s Goddard Earth Sciences Data and Information Services Center (GES DISC; https://disc.gsfc.nasa.gov/datasets/). This results in a latency of about three to seven weeks (barring re-running of the MERRA2 reanalysis by NASA or other operational interruptions). The archive starts on January 1, 1980, and will continue to grow over time as new MERRA2 driver data become available.

The data represent daily depths, in units of mm d^−1^, of reference evapotranspiration, denoted ETrs for a tall reference crop (0.5-m alfalfa), ETos for a short reference crop (0.12-m grass). The data are daily and global in extent at a resolution of 0.5° latitude and 0.625° longitude. The data are stored in NetCDF format, with one file for each day for each reference crop type; the daily data are also collected into annual NetCDF files for complete calendar years (i.e., 1980 to the last complete year). The daily netCDFs are compressed into zip files for each complete year and a single file for the period 1980 through the last complete year. The daily files run about 1.7 MB each and are named as follows: ETos_YYYMMDD.nc for short-crop ET_0_; ETrs_YYYYMMDD.nc for tall-crop ET_0_ (where YYYY is the 4-digit year, MM is the 2-digit month, and DD is the 2-digit day). The annual NetCDFs and zipped files run about 600 MB and 190 MB, respectively and are similarly named: ETos_YYYY.nc and ETrs_YYYY.nc for the annual NetCDF files; ETos_YYYY.zip and ETrs_YYYY.zip for the zipped files. The archives of all complete years run about 8.5 GB each and are named ETos_1980-ZZZZ.zip and ETrs_1980-ZZZZ.zip (where ZZZZ is the last complete year archived).

## Technical Validation

### Introduction

As the new ET_0_ reanalysis should address the limitations of the product it replaces, it requires verification against it; further verification is relative to station-based E_0_ observations from the region of greatest interest and to other global reanalyses. Following, we first summarise the verification against station-based data in southern Africa and then the verification against GDAS and other long-term, global reanalysis-based ET_0_ datasets. In “Usage Notes” we show a variety of case studies examining the utility of the new, MERRA2-driven ET_0_ dataset as an input to drought- and food security-monitoring operations.

### Station-based verification in southern Africa

In this section we describe the verification of the new MERRA2-based ET_0_ dataset against station-based observations derived from the SASSCAL network (www.sasscal.org)^30–32^. The ET_0_ values of the two data sets are compared both regionally and seasonally, using time series of station-based dekadal ET_0_.

**Fig. 1:**
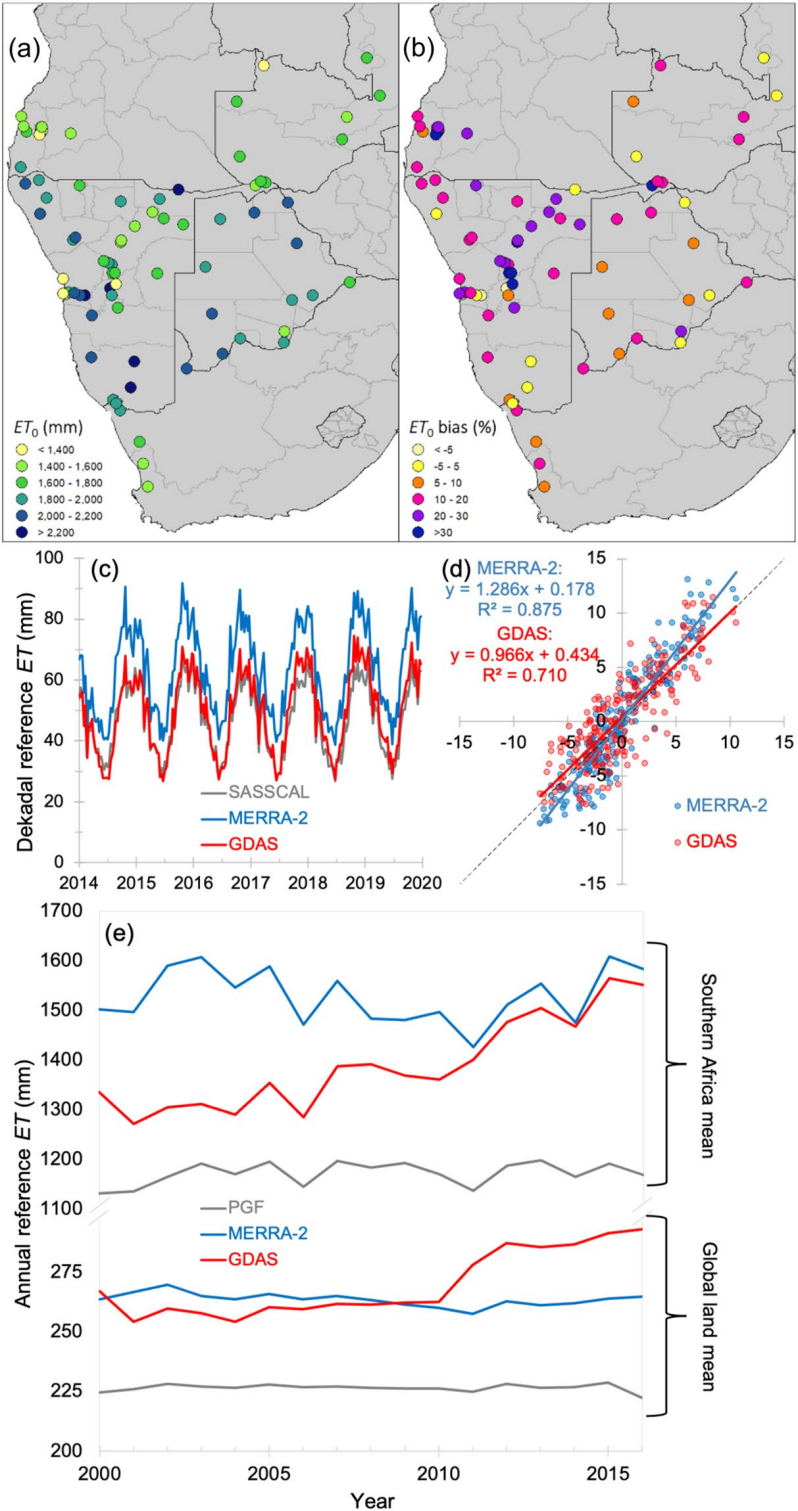
SASSCAL-observed ET_0_ compared to MERRA2- and GDAS-derived ET_0_. (**a**) Mean annual (2014–2020) ET_0_ estimated from SASSCAL stations across southern Africa. (**b**) Mean annual bias in mean annual ET_0_ derived from MERRA2 vs. SASSCAL (bias defined as the MERRA2 - SASSCAL difference as a percentage of SASSCAL). (**c**) Time series of dekadal ET_0_ from the (grey) 38 SASSCAL stations with the most consistent data availability, and (blue) the MERRA2 and (red) the GDAS reanalyses averaged over the Southern Africa region shown in (a). (**d**) Scatterplots of dekadal anomalies in (blue) MERRA2 and (red) GDAS against those in the SASSCAL stations, for the timeseries in (c). (**f**) Annual time series of areal mean ET_0_ driven by the (grey) PGF, (blue) MERRA2, and (red) GDAS reanalyses, over two regions: (top) the southern Africa region covered by the SASSCAL network, and (bottom) the global land area (note the discontinuous vertical axis).

Although limited spatially, with stations only in Angola, Botswana, Namibia, South Africa, and Zambia (see Fig. 1), the SASSCAL ET_0_ dataset is of high quality, with data derived from daily Automated Weather Station (AWS) observations for average air temperature, maximum air temperature, minimum air temperature, humidity, wind speed, barometric pressure, and solar irradiance. The daily data were summarised from stations with data loggers recording at 15-minute and 1-hour intervals. Some stations however had a number of sub-daily records, which would result in an inaccurate daily summary. To prevent inclusion of inaccurate daily data in the ET_0_ calculation, a quality control procedure removed stations reporting less than 83% of the expected daily records (a threshold minimum of 80 records per day for stations recording in 15-minute intervals, and 20 records per day for stations recording in 1-hour intervals). The 10-m wind speeds recorded at SASSCAL stations were adjusted to 2-m heights using a logarithmic wind profile^4^.

ET_0_ was calculated for a short-grass reference crop with a height of 0.12 m, a surface resistance of 70 s m^−1^, and an albedo of 0.23, as defined in the FAO-56 report^4^. The SASSCAL-based ET_0_ were aggregated to a dekadal timestep (i.e., 10-day totals) for the period 2014–2019. Only dekads with a maximum of one missing day of data were considered in the analysis. Only the 72 stations with full annual records (36 dekads) for one or more years were used in this analysis; the resulting dataset reports from 22 to 65 stations (average = 48.8) in each dekad across the 2014–2019 period, for a total of 10545 individual dekadal data points. The SASSCAL-derived ET_0_ data were then compared to data from the new NOAA ET_0_ dataset extracted directly at the SASSCAL station coordinates.

Figure 1 compares ET_0_ derived from the MERRA2 and GDAS reanalyses to that derived from SASSCAL observations across space and time in southern Africa. Panel (a) shows the mean annual ET_0_ for 2014 to 2019 at 72 SASSCAL stations in Angola, Botswana, Namibia, South Africa, and Zambia. In general, and as expected, ET_0_ is higher at stations in drier regions, such as the Namib and Kalahari deserts of Namibia and western parts of Botswana and South Africa, and lower at stations in wetter regions, such as Zambia. Panel (b) provides a spatially representative illustration of MERRA2-derived ET_0_ bias for all stations, with bias defined as ([MERRA2 or GDAS] – SASSCAL)/SASSCAL expressed as a percentage of SASSCAL). Across the population of all station-years, the bias ranges from +9.4% to +89.9%, with a mean of +36.1%, as MERRA2 ET_0_ in general exceeds SASSCAL ET_0_, increasingly so at stations in the northern half of Namibia, indicating overestimation of ET_0_ by MERRA2. GDAS ET_0_ bias is generally lower than MERRA2 ET_0_ bias. Performance of ET_0_ estimation by both MERRA2 and GDAS varied by country, though in almost all cases, variations in MERRA2-derived ET_0_ relates more closely to those in SASSCAL ET_0_ than variations in GDAS ET_0_ do. The lowest R^2^ for MERRA2-derived annual ET_0_ is for Zambia (0.36) and the highest for South Africa (0.93), where a good fit of the relationship is indicated. GDAS ET_0_ showed worse performance, with R^2^ ranging from 0.06 (Botswana) to 0.74 (Zambia); the single exception is Zambia, where the GDAS (R^2^ = 0.74) more closely relates to SASSCAL than MERRA2 does (R^2^ = 0.36).

Figure 1c,d compares time series of the MERRA2-based ET_0_ reanalysis to that based on GDAS; they are compared on a dekadal basis (i.e., ET_0_ totals for days 1–10, 11–20, and 20-month end for each month) relative to the data reported at the SASSCAL stations shown in Fig. 1a,b. For our comparison dataset, we selected all SASSCAL data for dekads during which all three data streams (GDAS, SASSCAL, and MERRA2) report data for over 66% of the available six-year period (2014–2019).

Clear in Fig. 1c is the positive bias (relative to SASSCAL) in MERRA2 ET_0_, which is clearly greater than that in the GDAS ET_0_. However, again, MERRA2 ET_0_ relate more strongly to the SASSCAL data than the GDAS data do. To demonstrate the relationship when the bias has been removed, Fig. 1d shows a scatter plot of the dekadal anomalies of both GDAS and MERRA2 plotted against the SASSCAL observational anomalies, where the respective mean dekadal values have been subtracted to remove the effects of seasonality and the bias. The correlation coefficient of MERRA2 is higher than that of GDAS (R^2^ of 0.88 for MERRA2, 0.71 for GDAS), though MERRA2 still overestimates ET_0_ relative to SASSCAL in higher ET_0_ conditions and underestimates in lower whereas GDAS does not (the slope of the relationship is 1.29 for MERRA2, 0.97 for GDAS).

This closer relationship of the MERRA2 ET_0_ indicates the positive benefits of bias-correction before use; anomalies (during droughts, say) are reflected better in the MERRA2 ET_0_ than in the GDAS ET_0_. While the positive bias in the MERRA2 indicates overestimation of observed data and could potentially lead to exaggeration of crop water demand, it should be noted that the time series of MERRA2 bias (not shown) was more consistent on an inter-annual basis. Standardised drought metrics use anomalies from mean driver conditions, so biases in the drivers are meaningless relative to the strength of the relationship of the metric to surface dryness conditions. Using MERRA2 ET_0_ data to model crop production (for example, with WRSI) without a bias correction might make crop performance appear worse and exaggerate water deficit during droughts. The results of this analysis suggest that the MERRA2 ET_0_ dataset can be used for applications in hydrology and agriculture because of its better correlation with observed data. However, it is recommended to implement adjustments or bias corrections to improve its suitability.

Further supporting the adoption of a bias-corrected MERRA2-based ET_0_ over the existing GDAS dataset is the fact that–while it appears that mean GDAS ET_0_ replicates the SASSCAL observations better than mean MERRA2 ET_0_ does–there is a non-homogeneity in the GDAS dataset that is not present in MERRA2. This non-homogeneity is shown in Fig. 1e: on or around 2010, annual mean GDAS ET_0_ averaged across the southern Africa region jumps by over 150 mm (a ~12% increase in the mean from 2000–2010 to 2011–2016, or 1333 mm to 1494 mm); the data in the analysis in Fig. 1c,d are drawn from the second period alone.

### Reanalysis-based verification across the globe

Here we summarise the verification of the new ET_0_ reanalysis against both the GDAS-driven ET_0_ dataset currently used by FEWS NET and ET_0_ derived from the PGF dataset, which is a primarily research-directed dataset (i.e., it is non-operational and has a high latency). These comparisons of ET_0_ of different drivers (GDAS, MERRA2, PGF, Table 1) should inform potential users of the dataset as to how it may differ from other established datasets and bracket uncertainty from reanalysis forcing. Other options may be available (e.g., CFS-R), which could be tested in the future but currently lie beyond our scope.

**Fig. 2:**
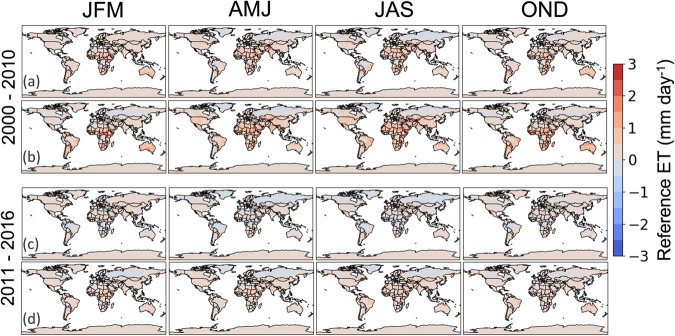
Average seasonal ET_0_ differences between MERRA2 and (**a,c**) GDAS and (**b,d**) PGF for: (**a,b**) the period 2000–2010; and (**c,d**) the period 2011–2016. As MERRA2 is the baseline, positive (negative) or red (blue) values indicate MERRA2 greater (less) than the comparison dataset.

Given that all three driver datasets have different native resolutions, we re-gridded all datasets to 0.125° using a bi-linear interpolation scheme in the NASA LIS software^33^ and averaged the sub-daily values to daily 2000–2016. The GDAS data shows a shift in the mean between 2007–2010 (Fig. 2), relative to both MERRA2 and PGF. The formulation of ET_0_ described in this paper was added to the NASA LIS software, specifically for the FEWS NET Land Data Assimilation System (FLDAS^34^), and was used to generate the ET_0_ estimates presented below.

Figure 2 shows that for the period 2000–2010, ET_0_ from MERRA2 exceeds that from GDAS [positive/red in panels (a) and (c)] across Africa and South America, though differences are relatively small (~1–2 mm d^−1^, 30–60 mm mon^−1^); differences from the PGF dataset are larger, and show some seasonal patterns (particularly in April and September), though in general the ET_0_ estimates are also lower than those from MERRA2 [by ~1–3 mm d^−1^; positive/red in panels (b) and (d)]. For the period 2011–2016 the bias between MERRA2-derived ET_0_ and GDAS-derived ET_0_ changes sign, with ET_0_ from GDAS higher than from MERRA2. This change begins in 2007, with the sign changing by 2011. This is potentially related to changes in GDAS interpolation scheme implemented in 2007, which would underscore the unsuitability of GDAS for driving long-term analyses. The MERRA2 vs PGF seasonal differences do not change (again, PGF-driven ET_0_ being lower than MERRA2-derived).

We find that, for ~90% of the global land-surface pixels, ET_0_ differences between products are less than 1 mm day^−1^. For seasonal averages (Fig. 2), the PGF-driven ET_0_ values are the lowest, MERRA2 ET_0_ the highest, with GDAS-driven ET_0_ producing values in the middle until 2010. From 2010–2016 we see ET_0_ from GDAS becoming larger than that from MERRA2 due to a shift in the GDAS mean (see Background and Summary and Fig. 1e). Similar patterns (both pre- and post-2010) are observed in month-by-month analyses (not shown). Consistent with our analysis, a comparison^34^ of monthly MERRA2, GDAS, and GLDAS temperature estimates over southern, eastern, and western African domains and found that all three products are well correlated (r > 0.7). However, GDAS has a notably low bias (about 1 °C) until ~2007 compared to MERRA2 and GLDAS/PGF temperature fields. From 2007-present, MERRA2 and GDAS temperatures continue to be well correlated and have a similar mean. In summary, the MERRA2 ET_0_ benefits from a more consistent record than the GDAS ET_0_, and produces robust magnitudes of ET_0_ in the context of two other reanalysis datasets.

## Usage Notes (applications)

The most common current uses of ET_0_ are in drought and agricultural monitoring. In this section, we examine examples of the uses of the new ET_0_ dataset in both of these fields. First, we place ET_0_ in the context of drought, and then examine the diagnosis and ongoing monitoring of drought, examining the phenomenon from the demand perspective. Next, we examine the utility of ET_0_ in supporting famine early warning in food-insecure countries where data are sparse, examining how this new ET_0_ dataset may be used to improve an important existing food-security monitoring tool, assist in seasonal forecasts, and inform climate-scale projections.

### Understanding and monitoring drought

The integration of advective and radiative forcing in evaporative demand (E_0_) and its strong physical coupling with actual ET^2,35^ make it a good metric of surface water availability and therefore of drought and crop water-stress^1^; while this is particularly true in regions of high advection or strong land-atmosphere coupling, E_0_ has been shown to be responsive outside of such regions^7^. Our ET_0_ provides a fully physical metric of E_0_ and therefore may serve as an independent driver of meteorological and agricultural drought metrics, particularly in regions where parameterizations of soil-water and plant-water availabilities are of questionable accuracy. These ET_0_ data can be used in estimating the Water Requirement Satisfaction Index (WRSI^10,36^), the Standardized Precipitation Evapotranspiration Index (SPEI^8^), the Standardized Evapotranspiration Deficit Index (SEDI^9^), and E_0_-based drought-monitoring and early warning tools such as the Evaporative Demand Drought Index (EDDI^6,7^), and in scientific analyses of the drivers of drought across the globe. More investigation will be required as to the extent that trends noted in MERRA2 and the associated ET_0_ data set presented here may influence both derived indices (e.g., EDDI, SPEI) that rely on climatology and anomalies (e.g., WRSI anomalies).

### Diagnosis of the demand side of drought

Drought is driven by a limitation of supply (i.e., precipitation) of moisture to the surface and/or an excess in demand (i.e., E_0_) for it in the atmosphere. This new ET_0_ reanalysis permits the first direct analysis of the demand side of drought in data-sparse areas of the globe. Here, we use the new ET_0_ dataset to determine the extent to which anomalies in ET_0_ (ΔET_0_) are due to each of its driver’s changes. Starting from the basic functional relationship between ET_0_ and its drivers, we see that these anomalies are related as follows:

7 $$\Delta E{T}_{0}=\frac{\partial E{T}_{0}}{\partial T}\Delta T+\frac{\partial E{T}_{0}}{\partial {R}_{d}}\Delta {R}_{d}+\frac{\partial E{T}_{0}}{\partial q}\Delta q+\frac{\partial E{T}_{0}}{\partial {U}_{2}}\Delta {U}_{2}$$

where the partial differentials are the sensitivities of ET_0_ to each of its drivers and the Δ operator denotes anomalies in ET_0_ (ΔET_0_) or its drivers (ΔX). Note that here we use a simplified expression for ET_0_, one that is expressed as a function of T (an average of T_min_ and T_max_) rather than of T_min_ and T_max_: this is for the sake of utility, as users are unlikely to benefit from the distinction. Sensitivity expressions are derived analytically from Eq. (1) for each driver X (where X = T, R_d_, q, or U_2_); these expressions are shown elsewhere^37^. Anomalies are derived from the reanalysis over the timescale concerned. Each of the four expressions on the right-hand side of Eq. (7) then represents the contribution to ΔET_0_ of the anomaly in a particular driver (in depth units). All anomalies are estimated daily, but should then be accumulated over more instructive timescales (examining daily anomalies would be very noisy), such as the elapsed period since the start of a growing season, water year, or drought event.

**Fig. 3:**
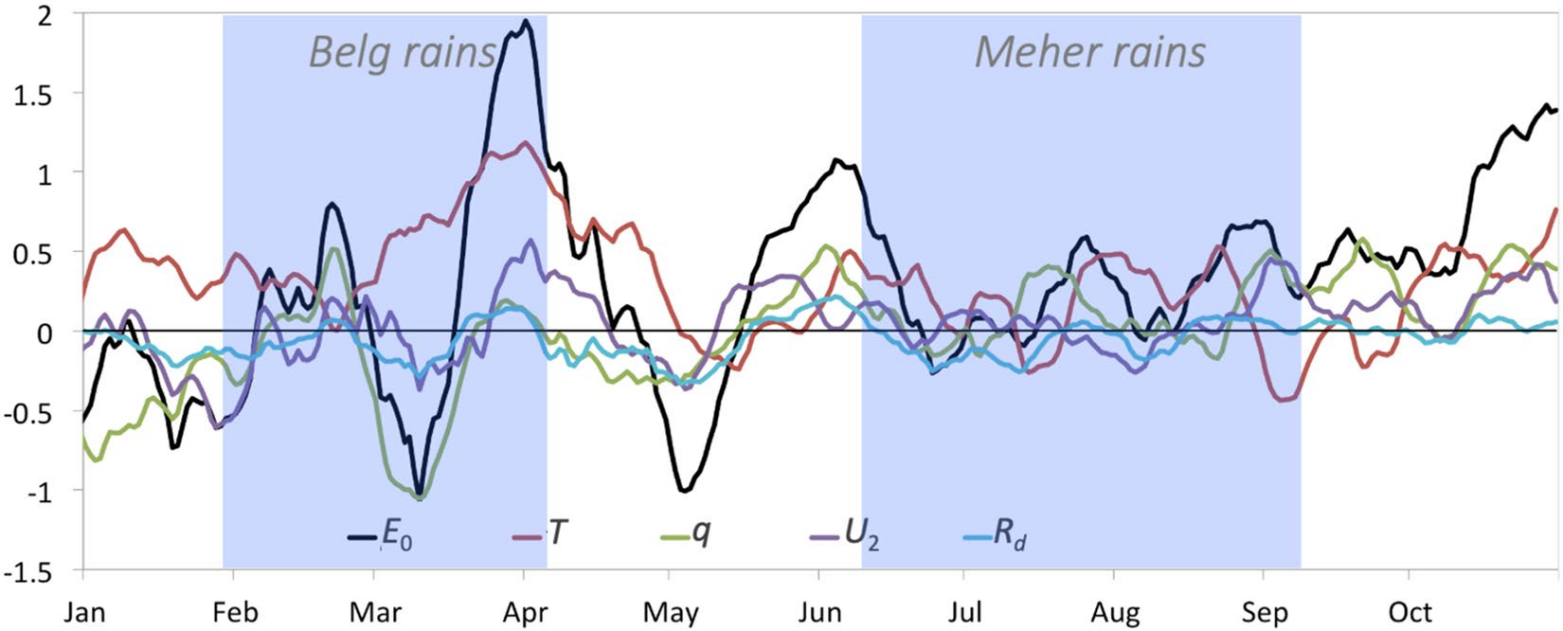
Time series of the contributions to changes in 10-day mean daily ET_0_ (mm depth; vertical axis) of changes in each of the drivers for the region shown in Fig. 4. The ET_0_ changes are indicated by the black line, and its constituent contributions from changes in T, q, U_2_, and R_d_ by the brown, green, purple, and blue lines, respectively. In all cases changes are shown relative to the long-term climatological mean (1981–2010) of the moving 10-day window (the zero-line).

**Fig. 4:**
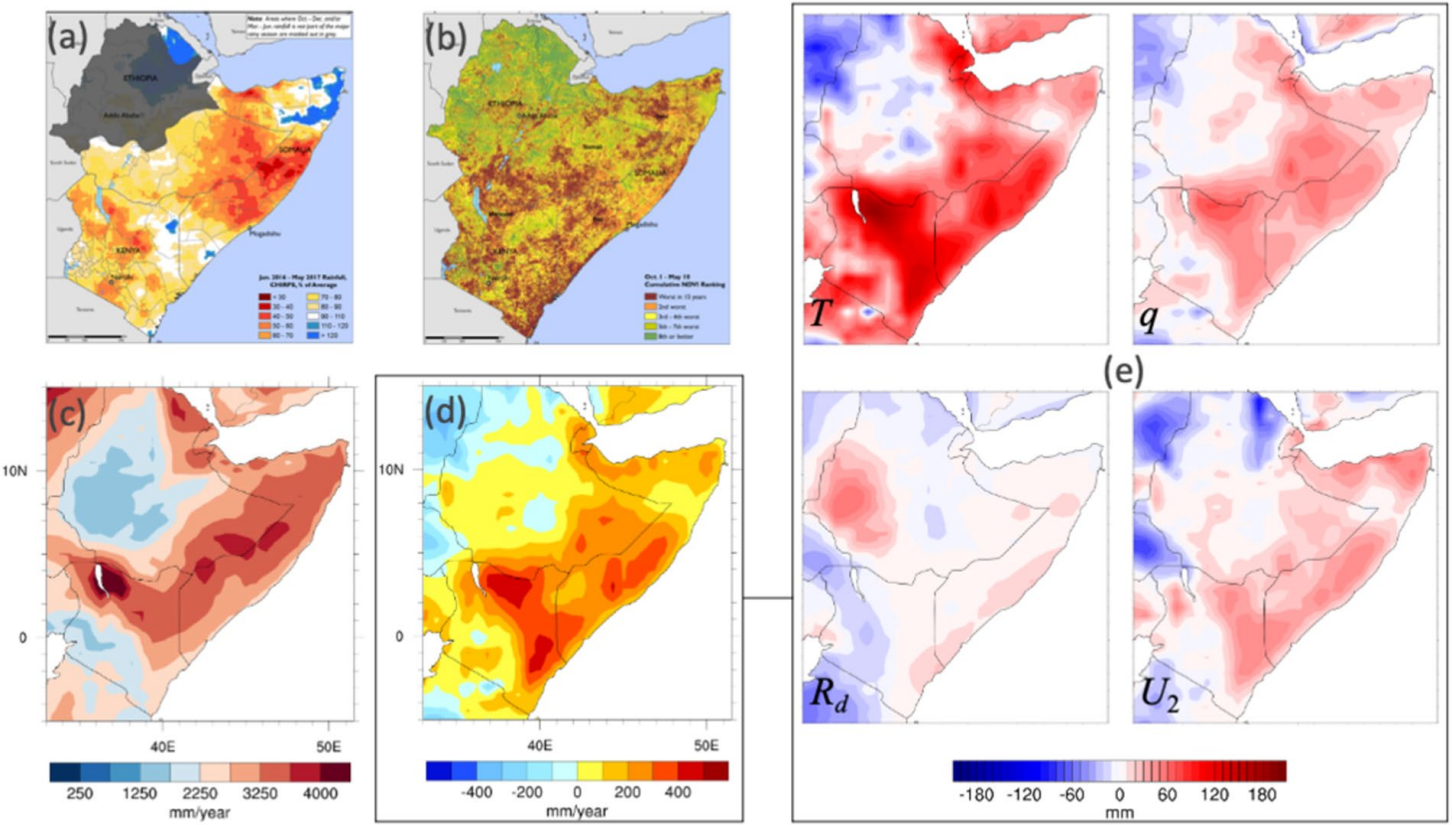
The 2016/2017 drought in the Horn of Africa represented by various metrics and decomposed as to drivers of evaporative demand (here ET_0_). Panel (**a**) shows the % of 30-year (1981–2010) mean annual precipitation estimated for June 2016 to May 2017; panel (**b**) shows the cumulative NDVI ranking for October 1, 2016 through May 10, 2017 (relative to the 14-year period ending in 2016); panel (**d**) shows the 12-month (June 1, 2016 to May 31, 2017) ET_0_ anomaly relative to the 30-year (1981–2010) mean annual ET_0_ shown in panel (**c**). Panel (**e**) represents the contributions in mm to the ET_0_ anomaly [in (**d**)] from each of its drivers, as labelled.

The accumulated ET_0_ anomaly and its relative contributions from each driver’s anomaly may then be plotted for a region as a whole to indicate what meteorological factors are driving the temporal development of the demand side of drought (in the case of positive ΔET_0_). This is shown in Fig. 3 for the 2016/2017 Horn of Africa drought. Here the anomalies in the drivers and in ET_0_ have been spatially averaged across the region (shown in Fig. 4). In the example of the 2016 Horn of Africa drought, changing ET_0_ was dynamically forced by elevated T, but peaks in ΔET_0_ coincide with elevated U_2_ and/or q minima, troughs with q maxima. R_d_ plays little role.

This decomposition can also be examined at each point (or pixel) across a region for a specific period of time to reveal regional patterns in the driving factors behind an acceleration in the demand side of the water balance and, by extension, of drought. This may then be presented in maps that clearly communicate these regional patterns. The 2016/2017 drought in the Horn of Africa is represented in Fig. 4 by various metrics and the decomposition of its demand side (i.e., ET_0_) into its radiative and meteorological drivers. The supply side of drought (precipitation) from June 2016 to May 2017 is shown as a proportion of climatological mean annual precipitation, with the most intense deficits shown in southern Somalia and eastern Ethiopia. The impacts on vegetation, shown as NDVI ranking (relative to a 14-year period) for the October 1 through May 10 period are also severe, and concentrated in Kenya and southern Ethiopia. There is a spatial mismatch between the vegetation impacts and the supply side drivers, with the worst impacts on vegetation located to the south and west of the greatest precipitation deficits. The explanation for this mismatch may lie in the demand side of this drought: the greatest excess in ET_0_ (the 12-month ET_0_ from June 1, 2016 to May 31, 2017 relative to the 1981–2010 mean) occurs across both regions.

Decomposing ΔET_0_ into contributions from each of its drivers shows that different drivers have different impacts on the demand side of drought, in terms of intensity and regionality: in the portion of the Horn of Africa covered by the precipitation map, most drivers are in agreement as to the direction and extent of the (positive, or drying) ΔET_0_; T appears to be by far the most significant driver, followed by q and U_2_ in broadly equal terms. Downwelling SW plays a relatively small role, except in a region of western Ethiopia somewhat removed from the epicentre of the drought; in western Kenya, it appears to be acting in an opposite direction (to reduce ΔET_0_) from the other drivers.

This analysis demonstrates that in some regions (e.g., in this drought, Kenya), the impacts of this drought were insufficiently explained by deficits in the supply side; in many regions, they were exacerbated by elevated E_0_.

This analysis further demonstrates the regional variability of drivers and impacts within a single drought, and that they may not be precisely co-located. Further, it shows that the drivers of the demand side of drought do not always work in concert, and themselves display distinct regional patterns. This new ET_0_ dataset lends itself to long-term analyses that would uncover whether these are drivers of drought in this region or responses to already dry conditions. If these drivers are predictable at long temporal or spatial scales, this would improve regional drought predictability.

### Evaporative Demand Drought Index (EDDI)

The Evaporative Demand Drought Index (EDDI^6,7^) is a recently developed ET_0_-based drought index that provides early warning and ongoing monitoring of agricultural and hydrologic drought and fire-weather risk. As EDDI is based on ET_0_ alone, it provides an ideal platform to demonstrate the utility of the new ET_0_ dataset in drought monitoring. The physical basis and derivation of EDDI are described elsewhere^6^, but, in summary, EDDI exploits the relationship between E_0_ and actual ET from the land surface, including two contrasting interactions. For one, there is a complementary interaction between ET and E_0_ in water-limited conditions whereby E_0_ (and hence ET_0_) responds to declining ET by increasing. There also exists a parallel relationship under energy-limited conditions, whereby increasing E_0_ forces ET higher and thereby reduces moisture availability. In all conditions, ET_0_ responds to drying by increasing, making it a robust indicator of drought, though these dynamics are particularly evident in regions of strong land-atmosphere coupling and/or strong advection. Due to the rapid response of ET_0_ to changes in land-surface moisture conditions and/or the forcing of them, EDDI is a leading indicator of drought. Relative to other established drought metrics across CONUS, EDDI holds particular promise in providing early warning of agricultural and hydrologic drought^7^. It has also shown promise in warning of incipient wildfire weather^38^.

Crucial to the accurate representation of the relationships between ET_0_ and ET that underpin EDDI is an accurate, fully physical measure of E_0_ that reflects all appropriate drivers (temperature, humidity, wind speed, and solar radiation) and that is distributed across the landscape of interest at an appropriate spatial scale and for long enough to derive a long-term climatology for the ranking process. The development of this fully physical, accurate, global, long-term ET_0_ reanalysis, covering equally well-metered and food-insecure regions of the world therefore presents an obvious opportunity for early warning of drought, famine, and incipient wildfire risk.

EDDI is estimated by first aggregating ET_0_ over a time window of interest (a potential drought-onset period, for example), and then ranking this aggregated E_0_ against the same period in all years of a long-term climatology. This rank is converted to percentiles of the standard normal (~N{0,1}) distribution, which are then categorised into levels of drought severity and mapped. Varying the length of the time window provides a multi-scalar drought index that can reflect drying processes that occur at different timescales.

**Fig. 5:**
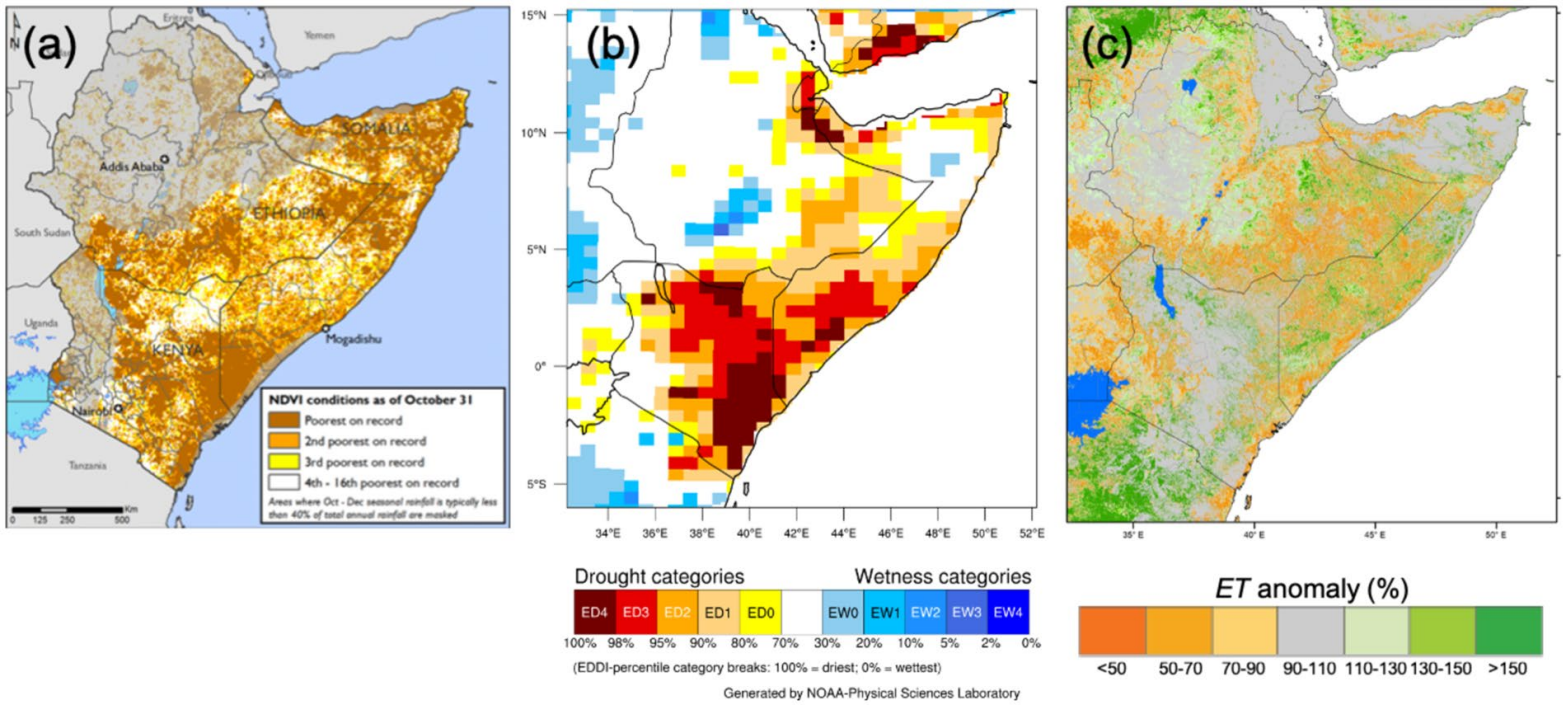
The 2016 drought in the Horn of Africa on October 31, 2016, as depicted by (**a**) NDVI (source: FEWS NET report), (**b**) 1-month EDDI, and (**c**) 1-month anomaly in actual ET.

The 2016 drought in the Horn of Africa is shown in Fig. 5, which displays conditions on October 31 as depicted by NDVI, the 1-month EDDI, and the 1-month ET anomaly (percentage of climatological mean). Epicentres of the vegetative impacts are observed in two primary regions—over Kenya and the southern tip of Somalia, and in the far-northern parts of Somalia and the eastern tip of Ethiopia—where NDVI denotes conditions as the poorest of the past 16 years. To a large degree, the 1-month anomalies in E_0_ (i.e., EDDI) and in actual ET flux (i.e., ET anomaly) are in broad agreement across space, particularly at the large scale—the Horn of Africa in drought and Tanzania wetter than normal. However, at the finer scale—looking at eastern Ethiopia, for example—there are differences. While these measures (EDDI and ET anomaly) should reflect the same hydroclimatic conditions, they are not the same fluxes and should not be expected to contain the same information: EDDI only reflects the demand for moisture in the atmosphere (i.e., E_0_), whereas the ET anomaly is also a function of the availability of surface moisture to meet this demand.

### Supporting food-security monitoring and famine early warning efforts

Here we examine the use of this new, global ET_0_ dataset in monitoring and projecting food insecurity for FEWS NET. These projections are based on likely food security scenarios that incorporate information on current conditions, assumptions of future conditions, potential changes to these assumptions, and possible behaviours of people in response to projected conditions. Food security scenarios depend on many factors that limit people’s access to food, including political conflict, health care, market prices, and environmental conditions. Environmental conditions are arguably the most important, given their close relationship with agricultural production.

FEWS NET crafts assumptions of environmental conditions relevant to agricultural production nine months into the future over five regions, including East Africa, West Africa, Southern Africa, Central America and the Caribbean, and Central Asia. Assumptions are developed in a monthly forum called the Seasonal Forecast Review, during which scientists from different disciplines provide evidence for likely future land surface, precipitation, and temperature conditions.

Strong environmental assumptions are based on a thorough understanding of the monitored state of the land surface and forecast conditions that directly impact the land surface. An example of an environmental assumption over East Africa may state, “Cropping conditions will be favourable during the October-December short rains over Kenya, Somalia and Ethiopia, given above average current land surface conditions and an above average forecast precipitation.” An assessment of current land surface conditions is based on daily ET_0_ and recent variations in ET_0_, among other variables that include soil moisture and NDVI. Forecast conditions are based on commingling the observed land surface state and predictions of precipitation and temperature from statistical and dynamical forecast models. Forecast land surface conditions are then used to advise the quality of cropping conditions and behaviour of agricultural yield.

### Water Requirement Satisfaction Index (WRSI)

**Fig. 6:**
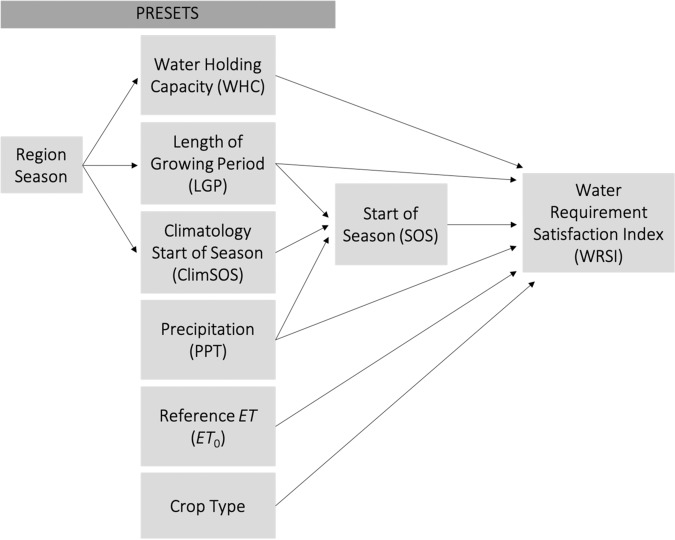
The GeoWRSI framework. In the 2nd column, the top three “preset” boxes refer to raster files containing information for seasonal water satisfaction calculations for the growing season and masked for each region. The lower three boxes represent the season-specific dekadal inputs and the crop type.

A tool commonly used by FEWS NET scientists monitoring and projecting crop stress is the Water Requirement Satisfaction Index (WRSI^39^): a leaky-bucket water-balance equation that estimates the amount of a plant’s water demand met by available water. The primary controls over this balance are the water coming into the system (via precipitation) and the demand for evaporation, or E_0_ (via ET_0_). The model was originally developed by the Food and Agriculture Organization (FAO)^39,40^ following two working papers on crop-water requirements^41,42^. Although the model was originally developed for implementation at a single site, the proliferation of gridded inputs allows for its implementation in a spatially explicit manner, using satellite-derived and otherwise spatially distributed drivers that make it ideal for application in data-sparse regions^10,11^. This is exemplified by the GeoWRSI version operational at FEWS NET and available through the USGS Early Warning data portal^43^. The GeoWRSI uses rasters containing preset season- and region-specific information on the Water Holding Capacity (WHC; used to account for runoff), the historical start of the season (ClimSOS; used to constrain when the start of the season can occur), and the length of the growing period (LGP; added to the SOS to identify the end of the season). Crop coefficients, which are available for a host of user-desired crop-types, are used to adjust the ET_0_ based on the phenology of different cereals and grain throughout the growing season^4,42^. Season-specific inputs of dekadal precipitation and dekadal ET_0_ are applied to the model. Once the season’s SOS is identified from precipitation thresholds, the WRSI is calculated for the length of the growing period (see Fig. 6).

However, this system has to date been constrained by its use of long-term arithmetic-mean climatologies (e.g., FAOCLIM) and meteorological inputs with a relatively short historical record [e.g., only since 2001 for NOAA Rainfall Estimate (RFE) precipitation and GDAS PET]. In contrast, the Climate Hazards group Infrared Precipitation with Stations dataset (CHIRPS^44^; available since 1981) and the dynamic, MERRA2-forced ET_0_ from NOAA provide a longer temporal record for ongoing monitoring and physically coupled water-balance calculations. In the following section, we compare the effects on analyses of seasonal water stress and food-security vulnerability when driven by the existing climatological ET_0_ relative to the new dynamic, MERRA2-derived NOAA ET_0_. We denote the climatologic dekadal ET_0_ (from GDAS) as “climatological ET_0_” and the ET_0_ dynamically varying in response to observed conditions (from MERRA2) as “dynamic ET_0_.”

### Using dynamic ET_0_ in growing-season water-stress forecasting

**Fig. 7:**
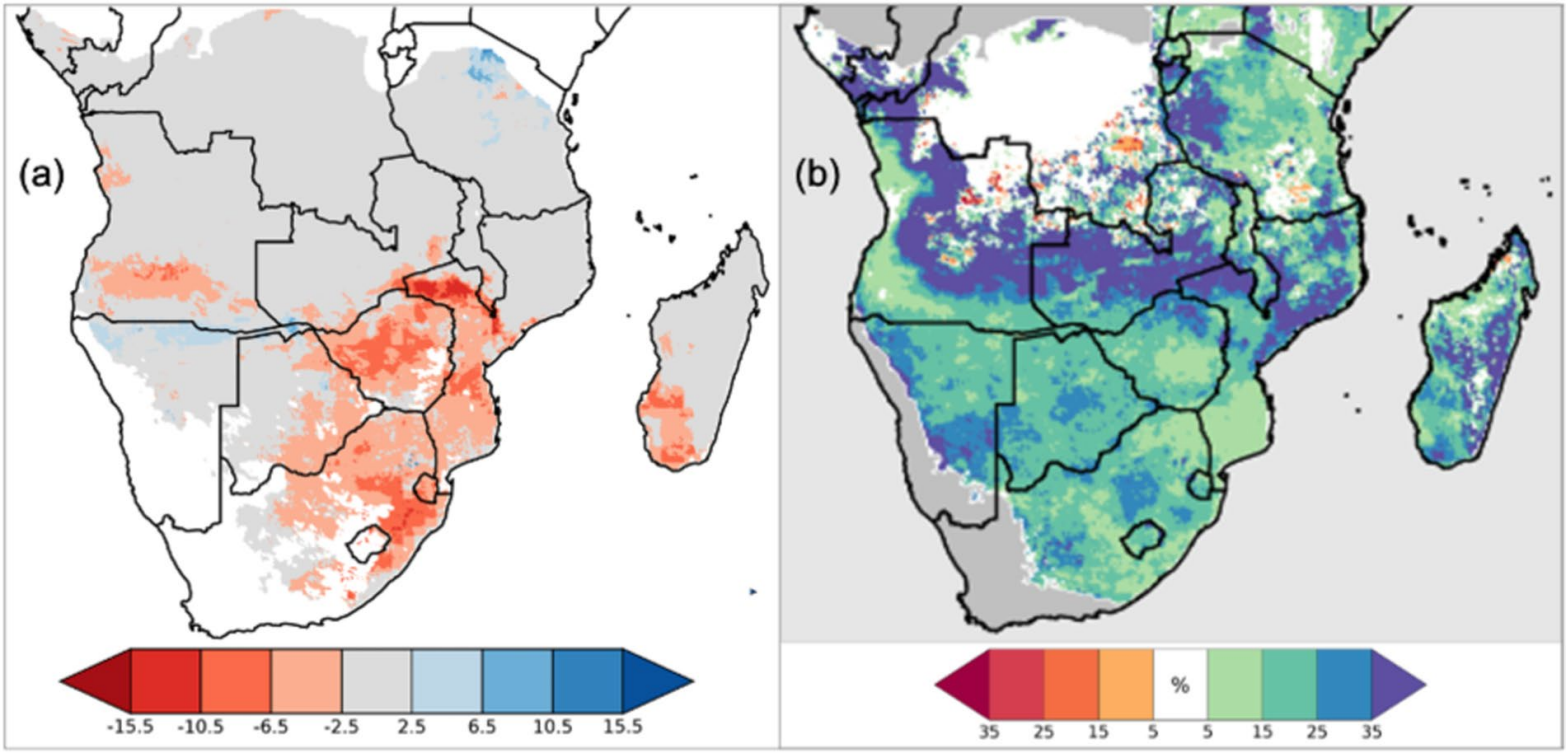
Comparing the effects on WRSI when driven by ET_0_ relative to that driven by climatological mean ET_0_. The left panel indicates the difference in 2015–16 end-of-season WRSI; blue (red) hues indicate relatively higher (lower) WRSI values when using the ET_0_, white indicates near-equivalent WRSI values. The right panel indicates the change in the standard deviation of historical (1981–2020) WRSI during regional growing seasons; blue (red) hues indicate higher (lower) variability of EOS WRSI when driven by ET_0_, white indicates near-equal variability. In both panels, grey indicates areas not normally included in WRSI analyses.

Relying on climatological ET_0_ has critical consequences in evaluating the impacts of drought, because reduced rainfall is often accompanied by increased E_0_, which can exacerbate water-stress. However, by definition, climatological ET_0_ cannot reflect this increased E_0_^45,46^. Figure 7 shows the impacts of ET_0_ choice—dynamic (from NOAA ET_0_) vs. climatologic (from GDAS-derived ET_0_)—in the WRSI model during the drought in the 2015–2016 growing season over southern Africa. One WRSI model run is forced by dekadal (i.e., 10-day totals) CHIRPS and dynamic ET_0_ from the 2015–16 season, and the other by dekadal CHIRPS (2015–2016) and a climatologic ET_0_, calculated as the dekadal arithmetic mean ET_0_ from 1981 to 2020. The drought occurred during an El Niño event, and was accompanied by above-average temperatures and increased ET_0_ over much of the region. Clear from Fig. 7 is how the WRSI driven by the elevated dynamic ET_0_ portrays a more severe drought and a greater crop-water deficit for the growing season across many of the agricultural regions of southern Africa, relative to the WRSI driven by climatologic ET_0_. More generally, using dynamic ET_0_ dramatically increases the variability of end-of-season WRSI, relative to WRSI run using climatologic ET_0_; doing so is crucial for putting present day events in historical context^47^.

Here, the primary advantage of this new, dynamic ET_0_ data set is that, combined with the CHIRPS precipitation data, the WRSI model can be run starting as far back as 1981 (the start of the CHIRPS dataset). This allows for the contextualization of conditions across a historical record that is twice as rich. With this new data set, FEWS NET will look to run the WRSI model operationally, incorporating this ET_0_ product, resulting in a better characterization of drought impacts on agriculture.

### Using dynamic ET_0_ in climate-scale projections

Following, we extend our analysis of the benefits of the new, dynamic ET_0_ dataset beyond growing-season forecasting to assessments of risk on multi-decadal timescales, examining an example from food-security vulnerability assessments in Africa, where FEWS NET has conducted satellite-based probabilistic agricultural drought-risk assessments since 2012^48^. This case study compares an analysis performed using dekadal values of the dynamic ET_0_ to one using a long-term, dekadal climatologic ET_0_, with precipitation inputs for both analyses coming from CHIRPS. The main objectives of these risk analyses are (i) to develop drought vulnerability functions for principal rainfed crops, (ii) to establish country-specific agricultural drought risk metrics, and (iii) to compare risk metrics for near-future climate scenarios (here, 2020 to 2040) relative to recent observed baseline periods.

**Fig. 8:**
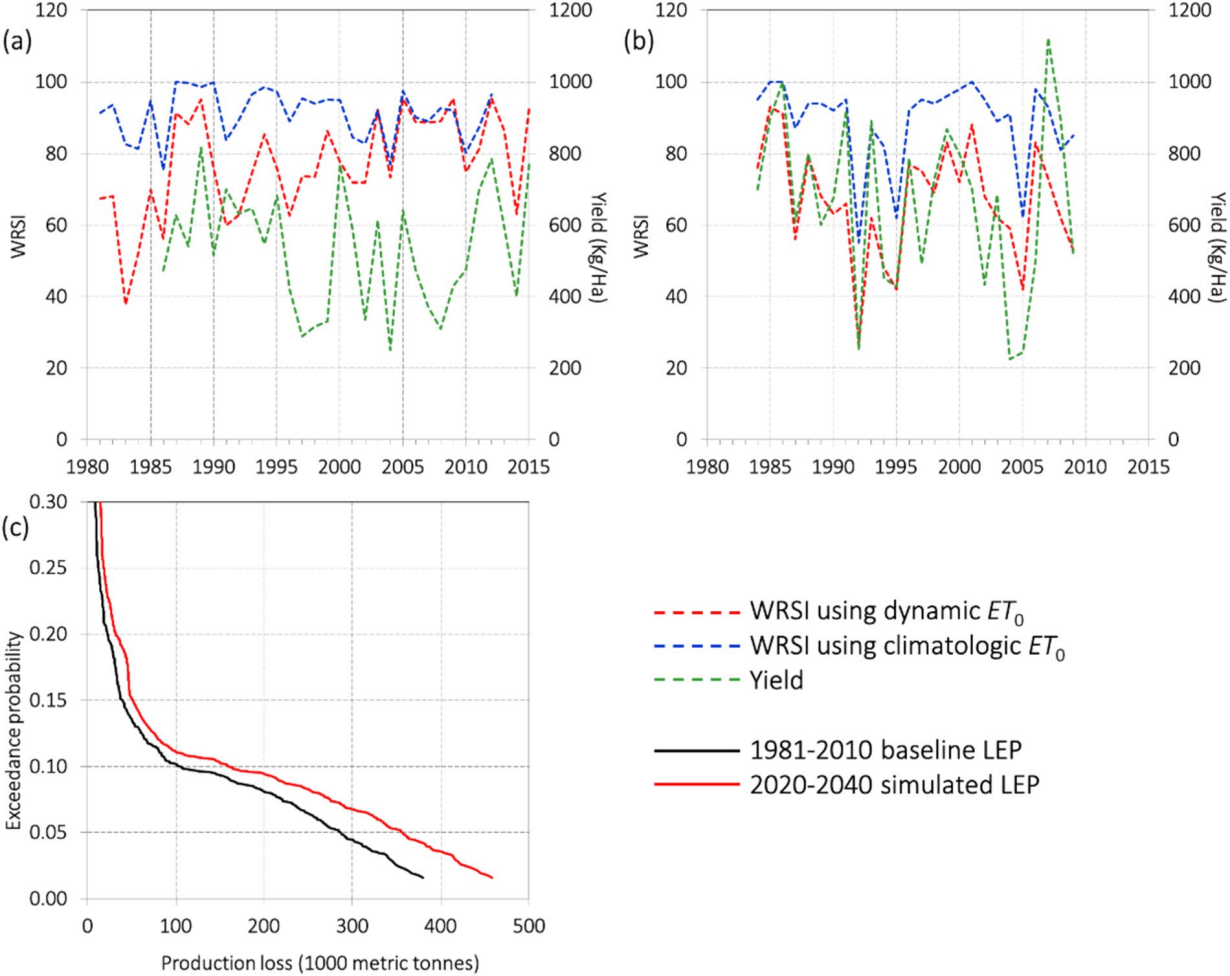
Using NOAA ET_0_ in agricultural drought-risk analysis. Panels (**a,b**) show WRSI time series for (**a**) millet in the Bambey district of Senegal and for (**b**) maize in Nsanje district of Malawi. Red (blue) lines indicate the WRSI time series derived using the NOAA ET_0_ (climatological mean ET_0_), green lines represent crop yields. In all cases, CHIRPS data is used for precipitation. Panel (**c**) shows the loss exceedance probability (LEP) curves for the baseline period (1981–2010) deduced from historical data (black line) and a near-future climate scenario (2020–2040; red line) for maize production (MT) in Malawi, both using CHIRPS and dynamic ET_0_.

Here we present the advantage of forcing the WRSI with precipitation from CHIRPS and dynamic ET_0_ in place of mean ET_0_ in the agricultural drought risk analysis. The initial phase of the study consisted of running GeoWRSI using CHIRPS precipitation and climatologic ET_0_ forcings to generate a WRSI time series, which in turn was used to generate agricultural drought risk metrics for principal rainfed crops in various regions in Africa^48–50^. Figure 8a,b shows WRSI time series generated using GeoWRSI forced by both climatologic ET_0_ and dynamic ET_0_ across two study areas and crops in Africa. The time series of crop yields are also shown to demonstrate the relative efficacies of using WRSI as yield proxies. In general, the following features stand out: (i) the use of dekadal CHIRPS and dynamic ET_0_ data beginning in 1981 enabled the inclusion of historical drought event losses of the 1980s and 1990s; (ii) although forcing the WRSI with climatologic ET_0_ works as a good first approximation, it is less sensitive for drought delineation than using dynamic ET_0_; (iii) dynamic ET_0_-forced WRSI identifies not only severe droughts but also mild droughts, whereas that forced by climatologic ET_0_ fails to detect mild droughts; (iv) long-term trends in the dynamic ET_0_-forced WRSI time series represent the changing climatic water supply and demand conditions better than that forced by climatologic ET_0_; and lastly (v) the millet and maize yields resonate better with dynamic ET_0_-forced WRSI than they do with WRSI forced by climatologic ET_0_. This last point illustrates the greater efficacy of WRSI forced by dynamic ET_0_ as a proxy for rainfed maize and millet yields. This comparative benefit is significant in developing effective WRSI-yield loss models^44^.

A time series of WRSI forced by CHIRPS and dekadal dynamic ET_0_ was also used to estimate the drought-induced maize production losses in a probable near-future climate scenario using the loss exceedance probability (LEP) curve approach. LEP curves are essentially continuous probability distribution curves constructed using rank-ordered historical and simulated event losses. Figure 8c demonstrates the LEP curves for a baseline period (1981–2010) and a probable near-future climate scenario (centred at 2030) using corresponding long-term synthetic precipitation and dynamic ET_0_ data^51^. The X-axis indicates drought-induced maize production losses while the Y-axis indicates the frequency of drought expressed in terms of exceedance probability (EP). The impact of a one-in-a-20-year drought is more severe than that of a one-in-10-year drought. A comparison of the above profiles clearly indicates that maize production losses will increase in the near-future changed climate, and that the magnitude of maize production losses will increase exponentially reducing EP, i.e., more severe droughts are projected in Malawi.

## Data Availability

The ET_0_ data are derived using approximately 1,000 lines of code written in bash scripts and NCAR Command  Language (NCL). While not written as a portable library, access to the code is not restricted, and it is available for download from USGS Sciencebase (10.5066/P9IIQMV1).
